# Current status of the clinical use of PD-1/PD-L1 inhibitors: a questionnaire survey of oncologists in China

**DOI:** 10.1186/s12885-020-6583-3

**Published:** 2020-01-31

**Authors:** Bicheng Zhang, Yuxiao Song, Yang Fu, Bo Zhu, Baocheng Wang, Jun Wang

**Affiliations:** 1 Cancer Center, Renmin Hospital of Wuhan University, Wuhan, 430060 China; 2 Department of Oncology, Xiangyang Hospital, Hubei University of Chinese Medicine, Xiangyang, 441001 China; 3 Cancer Institute, Xinqiao Hospital, Army Medical University, Chongqing, 400037 China; 4 Department of Oncology, No. 960 Hospital of People’s Liberation Army, Jinan, 250031 China; 5 Department of Oncology, The First Affiliated Hospital of Shandong First Medical University, No. 16766, Jingshi Road, Jinan, 250014 China

**Keywords:** PD-1/PD-L1 inhibitors, Current status survey, China

## Abstract

**Background:**

The purpose of the present study was to obtain information on the use of PD-1/PD-L1 inhibitors by oncologists in China through a national questionnaire survey.

**Methods:**

Between the 7th and 25th of July in 2019, a questionnaire designed by the Chinese Society of Clinical Oncology Immuno-Oncology (CSCO IO) Committee on the current status of the use of PD-1/PD-L1 inhibitors was distributed online and offline to cancer-related medical departments in thirty different provinces and autonomous regions of China. The national questionnaire consisted of three sections as follows: general information, current status of the application of PD-1/PD-L1 inhibitors in the clinic, and oncologists’ concerns regarding utilization.

**Results:**

The valid response rate of the current status survey was 76.3%. The proportion of senior doctors (physician-in-charge or a more superior position for more than 3 years) among the respondents was relatively high (67.0% in 588). Of the respondents, 59.2% had prescribed PD-1/PD-L1 inhibitors during clinical treatment, and the most frequent reason for not prescribing these inhibitors was the choice “do not understand the mechanism and the efficacy of PD-1/PD-L1 inhibitors”. In addition, 77.9% of the prescribers used the medications in an off-label situation, and the most important motivation for this use was the fact that “there were indications abroad but not domestically”. In addition, 77.9% of the prescribers believed that “immunotherapy-related adverse effects could be controlled or intervened through follow-up management”. The prescribers were mostly concerned about “how to identify pseudoprogression and hyperprogression” and “immunity-related adverse effects management”.

**Conclusion:**

The present study highlights the current status of PD-1/PD-L1 inhibitors in China. Increasing numbers of medical oncologists are interested in PD-1/PD-L1 inhibitors, and they are in need of immunotherapy education.

## Background

Immunotherapy has recently arisen as an attractive and feasible alternative treatment method for a subgroup of patients with various cancer types. This therapeutic strategy, which works by enhancing the function of antitumor T lymphocytes, is especially promising and has yielded exceptional results in clinical cancer treatment [1]. Based on so many successful experiences, there is now optimism that ongoing endeavors in the field of tumor immunology will further promote the efficacy of this groundbreaking cancer treatment, which has already resulted in many sustained remissions in a subset of cancer patients [1]. The transition of immunotherapy from a theory to standard-of-care therapy has been driven by years of work. In particular, immune checkpoint inhibitor (ICI) therapies based on monoclonal antibodies have been proven to be remarkably effective [2].

Cytotoxic T lymphocyte-associated protein 4 (CTLA-4) was the first negative regulator of T cell activation to be identified in 1994, and it is an inhibitory receptor upregulated early in the course of T cell activation [3]. CTLA-4 has become a potential therapeutic target aiming to strengthen the activity of effector T lymphocytes in the course of T cell activation. Based on encouraging data from a phase III trial on metastatic melanoma, the first CTLA-4 checkpoint inhibitor, ipilimumab, was approved by the Food and Drug Administration (FDA) in 2011 [4]. Despite being approved for only unresectable or metastatic melanoma, ipilimumab can be viewed as a promising therapeutic strategy for many cancer types, such as renal cell carcinoma (RCC) [5], non-small cell lung carcinoma (NSCLC) [6] and small cell lung cancer (SCLC).

Several years after the discovery of CTLA-4, programmed cell death 1 (PD-1) was identified as a coinhibitory receptor that negatively regulates the function of effector T lymphocytes [7]. In addition, programmed cell death ligand 1 (PD-L1) was identified as a ligand for PD-1 [8]. As many studies have shown, maintenance of the effector phase of antitumor T lymphocytes relies on blockade of the PD-1/PD-L1 checkpoint. Based on the accumulating data, the FDA approved a monoclonal antibody, nivolumab, for unresectable or metastatic melanoma in 2014 as a second-line therapy [9]. In addition to melanoma, indications for nivolumab approved by the FDA include squamous/nonsquamous NSCLC, advanced RCC, classical Hodgkin lymphoma, recurrent squamous cell carcinoma of the head and neck (SCCHN), advanced or metastatic urothelial carcinoma and advanced hepatocellular carcinoma (HCC). More recently, another PD-1 checkpoint inhibitor, pembrolizumab, has attracted much public attention. Originally, pembrolizumab was granted approval as an alternative to nivolumab for second-line treatment of patients with unresectable or metastatic melanoma [10]. Other indications of pembrolizumab that were later approved by the FDA include metastatic NSCLC, classical Hodgkin lymphoma, SCCHN, urothelial carcinoma, gastric/gastroesophageal junction adenocarcinoma and colorectal cancer.

In August 2018, nivolumab and pembrolizumab were approved by the China Food and Drug Administration (CFDA) as therapies for locally advanced or metastatic NSCLC and unresectable or metastatic melanoma, respectively, while ipilimumab had not yet been used in China. Since the end of 2018, three PD-1 inhibitors made in China (toripalimab, sintilimab, and camrelizumab) [11, 12] have entered the domestic market. However, Chinese doctors generally lack a basic knowledge of immunotherapy, and there are phenomena such as abuse, contraindications, and use of off-label medication in clinical practice. Therefore, investigating the current status of immunotherapy in cancer-related medical departments in China is useful. The purpose of the current survey is to collect clinical information on the usage of different PD-1/PD-L1 inhibitors in China, which would help Chinese oncologists standardize clinical practice when applying PD-1/PD-L1 inhibitors. This questionnaire was designed and initiated by the Immuno-Oncology (IO) Committee, Chinese Society of Clinical Oncology (CSCO).

## Methods

This questionnaire was initiated and designed by the CSCO IO Committee. Between the 7th and 25th of July in 2019, a questionnaire regarding the current status of use of PD-1/PD-L1 checkpoint inhibitors was distributed online and offline to the cancer-related medical departments in thirty different provinces and autonomous regions of China. This questionnaire consisted of three sections as follows: general information, made up of three questions; status of application of PD-1/PD-L1 inhibitors in the real world, involving four questions; and doctors’ concerns regarding utilization.

In the general information section, questions related to the prescribing physicians’ origins included the doctors’ locations and professional qualifications, as well as the hospital levels and clinical departments they worked in. In the second part of the questionnaire, the underlying medical picture surrounding prescription of the PD-1/PD-L1 inhibitors, the reason for off-label medication, the personal countermeasures taken when the therapy was found ineffective, and the prescribing physician’s personal level of knowledge of the drug were requested. In the section regarding the doctors’ concerns regarding utilization, physicians were requested to identify the issue that concerned them most.

## Results

A total of 771 questionnaires were collected in this survey, 588 of which were identified as containing valid answerers provided by clinicians after filtration. Thus, the valid response rate of the current status survey was 76.3%.

### General information

The survey was an investigation of a large number of doctors of various professional qualifications nationwide from different types and cancer treatment-related departments of hospitals in thirty provinces and autonomous regions of China.

According to statistical analysis, we sorted the amount of the questioned doctors from different locations in decreasing order as follows: Shandong Province (105, 17.8%), Hebei Province (86, 14.6%), Guangdong Province (70, 12.0%), Henan Province (48, 8.2%) and Jiangsu Province (37, 6.3%).

Among the surveyed doctors, 588 were divided into 6 categories based on their professional qualifications. There were 82 chief physicians (13.9%), 153 associate chief physicians (26.0%), 159 physician-in-charge-level physicians for more than 3 years (27.0%), 71 physician-in-charge-level physicians for less than 3 years (12.1%), 106 resident physicians (18.0%) and 17 physicians with other qualifications (2.9%).

The responding doctors came from different types of hospitals that varied in hospital levels, consisting of five levels: 341 doctors came from grade III level A hospitals (58.0%), 59 came from grade III level B hospitals (10.0%), 106 came from grade II level A hospitals (18.0%), 76 came from cancer hospital (12.9%), and 6 came from other institutions (1.0%).

We also surveyed the responding physicians’ distribution across a diverse range of cancer treatment departments within the hospitals, including 365 doctors from medical oncology departments (62.1%), 64 from tumor-related surgical departments (10.9%), 65 from hematology departments (11.0%), 41 from radiotherapy departments (7.0%), 18 from chemoradiotherapy departments (3.1%) and 35 from other departments (6.0%).

### Status of the application of PD-1/PD-L1 inhibitors in the clinic

#### Prescription

**Fig. 1:**
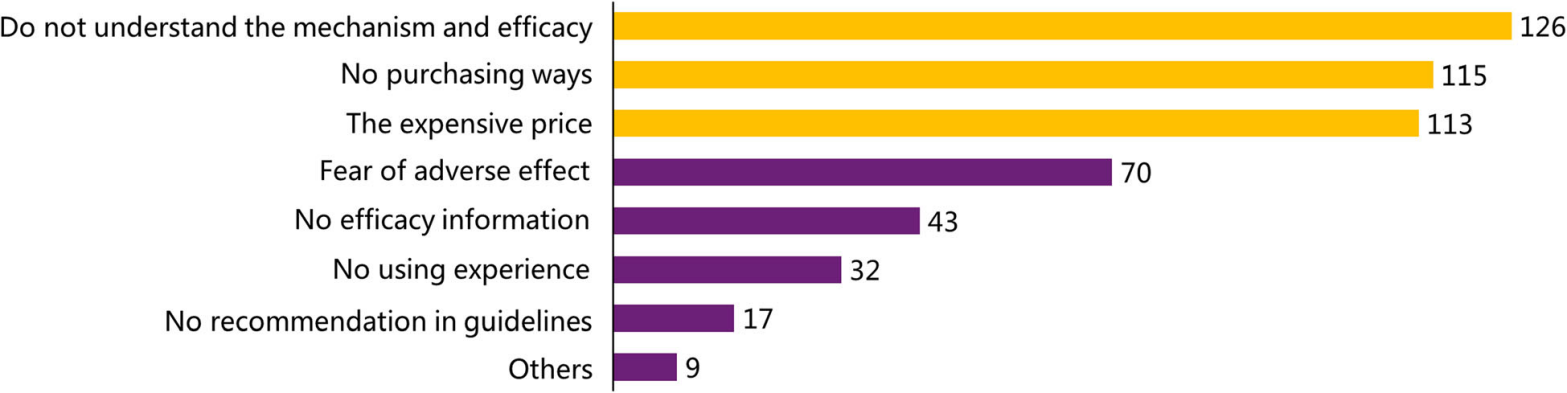
The results of the question “What is your reason for not prescribing?”

According to the survey, less than one-third (158, 26.9%) of respondents participated in clinical trials, while the rest (430, 73.1%) did not, but 348 of the 588 respondents, approximately 59.2% of the doctors surveyed, had prescribed PD-1/PD-L1 inhibitors during clinical treatment. The 240 doctors who did not prescribe PD-1/PD-L1 inhibitors were asked to choose from several reasons listed for why they had not prescribed these inhibitors, which suggested three major reasons, including 126 responses for “do not understand the mechanism and the efficacy of the drug”, 115 responses for “no purchasing options” and 113 responses for “the expensive price”. The results are presented in Fig. 1. The number of responses for each option was calculated and the results are displayed in order from highest number of responses to lowest number of responses. The most common reasons are highlighted in yellow. When the doctors were asked the question “Would you choose the imported (eg. nivolumab and pembrolizumab) or domestic drugs (such as toripalimab, sintilimab, and camrelizumab) in China as the preferred alternative?”, 44.8% of them answered “unbias”, 35.1 and 20.1% of the doctors said that they would select imported or domestic drugs firstly, respectively.

#### Indication for medication

In this survey, PD-1/PD-L1 inhibitors were shown to have been applied in many tumor types, and their application in lung cancer far exceeded that in other tumor types. The order of application for PD-1/PD-L1 inhibitors in different cancer types ranging from highest use to lowest use is as follows: lung cancer, liver cancer, melanoma, colorectal cancer, lymphoma, gastric cancer, esophageal cancer, renal cancer, and breast cancer. The survey showed that 271 (77.9%) of the 348 prescribers used the inhibitors in an off-label situation, and of these, 115 physicians (33.0%) indicated that they used the inhibitors in off-label situation in “1-9% of cases”, 83 physicians (23.9%) indicated that they used the inhibitors in off-label situations in “10-20% of cases”, 45 physicians (12.9%) indicated that they used the inhibitors in off-label situations in “30-50% of cases”, and 28 physicians (8.0%) indicated that they used the inhibitors in off-label situations in “over 50% of cases”.

**Fig. 2:**
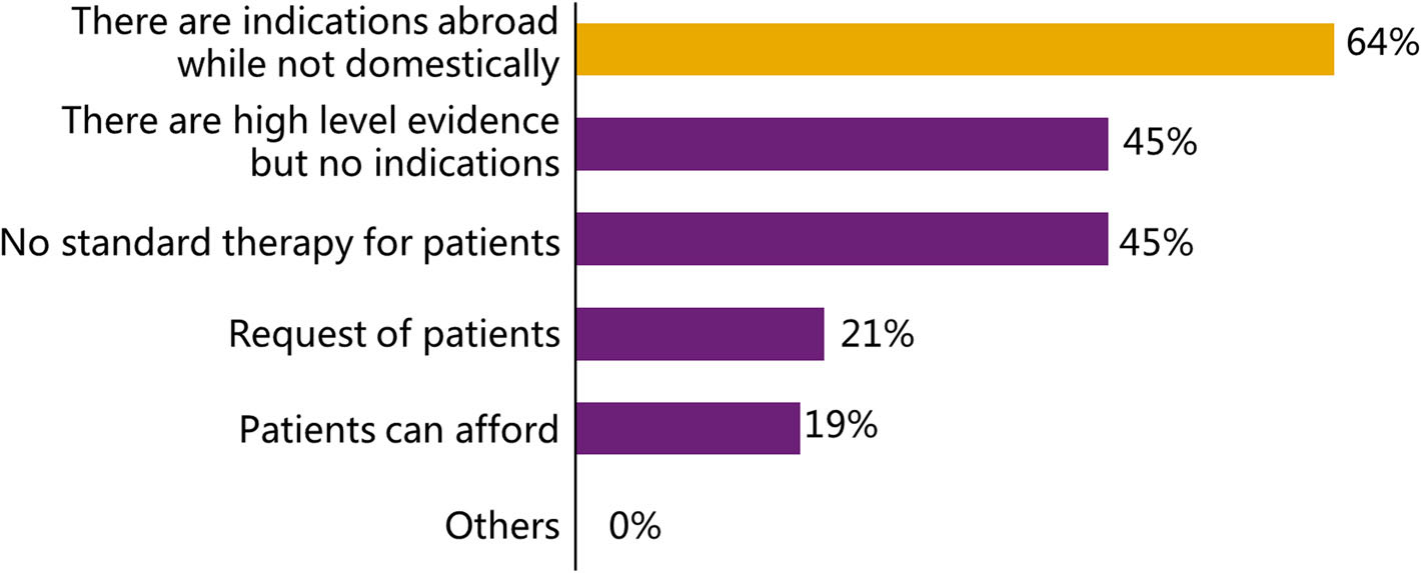
The results of the question “What is your motivation for off-label medication?”

**Fig. 3:**
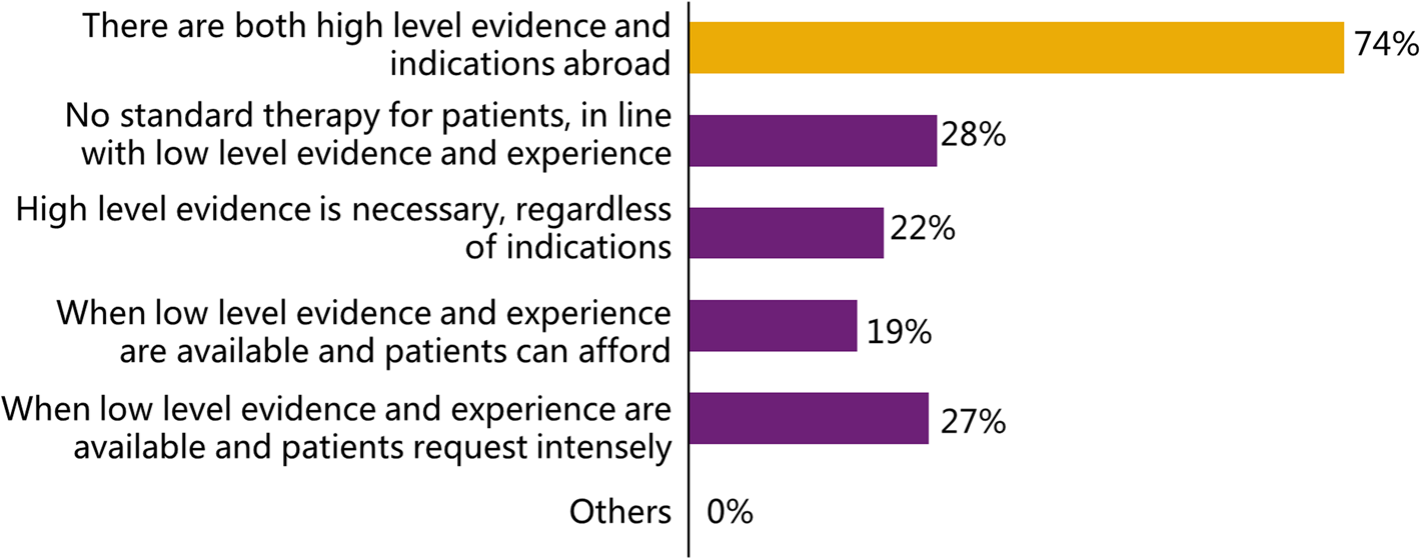
The results of the question “What is your criterion for off-label medication?”

The motivation for off-label medication was surveyed in the questionnaire, and the 271 “experienced” doctors were asked to choose their motivation. The most important motivation was that “there are indications abroad but not domestically”, which was chosen by 173 doctors (63.8%) of 271. Additionally, the criteria for carrying out off-label medication were surveyed as well. Most doctors (200 of 271, 73.8%) agreed that there had to be both advanced evidence and overseas indications. The detailed results of the motivation survey are presented in Fig. 2. The proportion of prescribers for each option was calculated, and the most important motivation is marked in yellow. In Fig. 3, the detailed results of the criteria survey are shown; the prescribers used off-label medication based on diverse criteria, and the most common criterion is highlighted in yellow.

#### Personal countermeasures when the therapy was found ineffective

**Fig. 4:**
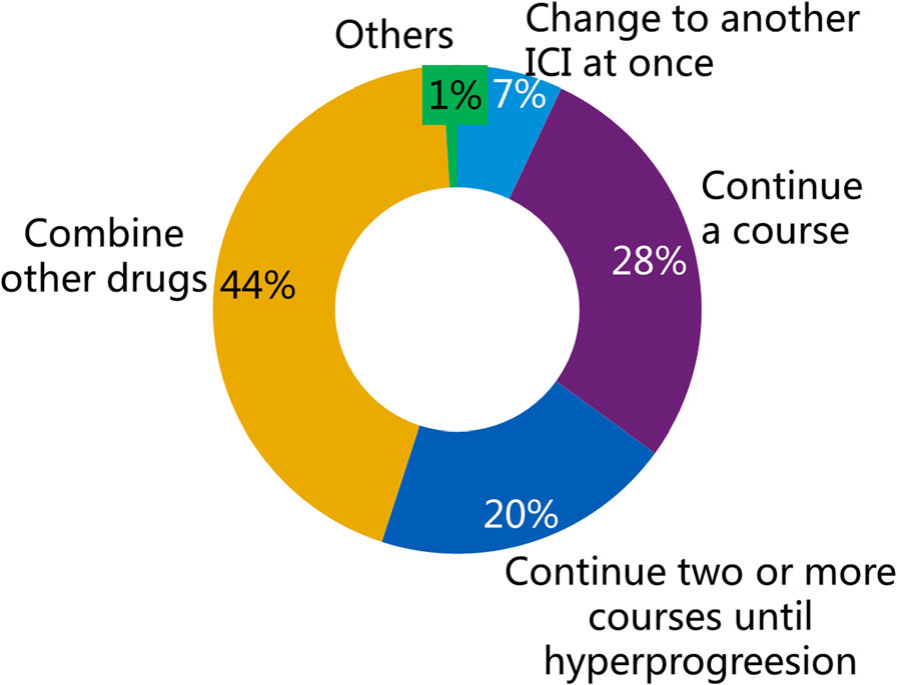
The results of the question “What is your countermeasure when the therapy is found ineffective?” This question was answered by all 588 respondents

Although some respondents did not prescribe PD-1/PD-L1 inhibitors, all 588 physicians were required to answer this question. They had to choose a countermeasure when the efficacy of the drug was not observed at an early stage of immunotherapy, and the majority (259 doctors, 44.0%) of them indicated that they would consider a combination with other drugs. The details of the personal countermeasures survey are displayed in Fig. 4, which shows that most respondents would consider continuing the ongoing PD-1/PD-L1 inhibitor therapy in several ways. In addition, when asked whether they would change to another PD-1/PD-L1 inhibitor if such an event occurred, 66.0% of 588 respondents chose “No”, and the remaining 34.0% chose “Yes”.

#### Prescribing physician’s knowledge of drug safety

The 348 prescribing physicians ultimately shared a clear and general consensus on the indications for withdrawal of PD-1/PD-L1 checkpoint inhibitors, with the survey indicating that the drug needed to be discontinued when grade III and above adverse effects (257, 73.9%) or hyperprogression (223, 64.1%) occurred. Most doctors (264 of 348, 75.9%) held the perspective that immunotherapy-related adverse effects could be controlled or managed through follow-up management. In contrast, a few doctors (17, 4.9%) claimed that the drug should be applied cautiously due to the occasional occurrence of serious adverse effects. Additionally, some doctors (66, 19.0%) believed that training about adverse effects of treatment should be strengthened.

### Oncologists’ concerns regarding utilization

When the 348 prescribing physicians were asked “Is it necessary to detect the gene expression level of PD-L1 before PD-1/PD-L1 inhibitor therapy?”, 212 (60.9%) chose “Yes”, 21 (6.0%) chose “No”, 97 (27.9%) chose “Judge according to drug indications”, 14 (4.0%) chose “Not sure”, and 3 (0.9%) chose “Other”. However, when they were asked, “Is it necessary for patients to do the large-panel gene detection before PD-1/PD-L1 inhibitor therapy?”, 118 (33.9%) chose “Yes”, 111 (31.9%) chose “It depends, and I know clearly whether a patient needs it or not”, 76 (21.8%) chose “It depends, but I am not very sure whether a patient needs it or not”, 38 (10.9%) chose “Generally, no”, and 4 (1.1%) chose “I do not know”.

What the 348 doctors using the PD-1/PD-L1 inhibitors were most concerned about was also included in the questionnaire. The results showed four groups of answers, including two identical groups (both included 111 of 348, 32%) of doctors who were most concerned about “how to identify pseudoprogression and hyperprogression” and “immunity-related adverse effect management”, 66 doctors (19.0%) who were concerned about “how to further improve the efficacy of the drug”, and 59 doctors (17.0%) who were concerned about “resistance mechanism and selection of follow-up treatment”.

## Discussion

This national survey is the first to investigate the current status of PD-1/PD-L1 inhibitors in the Chinese mainland by distributing a questionnaire to physicians involved in cancer treatment. Among the clinicians who returned valid questionnaires, nearly 60% had prescribed PD-1/PD-L1 inhibitors, which indicates that Chinese clinicians are actively exploring the best role for immunotherapy in patients suffering from cancer in China.

In terms of the distribution of respondents’ location, 58.9% of them were located in eastern provinces in China. Because the economy in eastern China develops faster than that in the west, naturally, doctors from eastern provinces are more likely to communicate with the international community regarding medical topics. With respect to the professional level of the survey respondents, they were from various high-level hospitals in China. Therefore, the respondents of the survey are representative of the all doctors involved in cancer diagnosis and treatment. Additionally, the proportion of senior doctors (physician-in-charge or a more superior position for more than 3 years, 66.9% in total) among the clinicians participating in the survey was relatively high, so the results of the survey are representative of the overall outlook regarding immunotherapy of tumors in the Chinese mainland. However, aside from the doctors (83.1% in total) from cancer-related departments, there was a considerable proportion (11.0%) of doctors from hematology departments, which might be attributable to the numerous recent advances in immunotherapy for treatment of hematological tumors [13].

In actuality, most (59.2%) clinicians that were surveyed had already prescribed PD-1/PD-L1 inhibitors in clinical work, while only a few (26.9%) of the prescribing physicians ever taking part in ICI-related research, including clinical trials. These results can be interpreted in two ways.

On one hand, the fact that PD-1/PD-L1 inhibitors have wide application across various tumors reflects their theoretic validity, which has been recognized by an increasing number of Chinese prescribers. Considering treatment of NSCLC as an example, in just 3 years, immunotherapy has progressed from monotherapy to combined therapy, from second-line standard therapy for advanced tumors to first-line therapy [14] and from salvage therapy for advanced tumors to consolidation therapy for locally advanced tumors and neoadjuvant therapy for early and mid-stage tumors [15]. The rapid accumulation of evidence of the efficacy of PD-1/PD-L1 inhibitors has inspired Chinese physicians to implement them in practice. Weiss et al recently proved that pembrolizumab could be given to metastatic pancreatic adenocarcinoma patients, and the efficacy appears to be improved compared with that of standard gemcitabine and nab-paclitaxel dosing [16]. In fact, similar combination immunotherapy has been applied as a first-line treatment for advanced pancreatic cancer in many hospitals in the Chinese mainland. In addition, increased use of the drug can stimulate medical departments to carry out ICI-related research in line with the specific characteristics of Chinese cancer patients, which probably will enable the medical staff to more precisely diagnose and cure ‘immunity-related adverse effects’, which were noted as one of the top two concerns of the prescribers on the survey. Of note, the CSCO IO Committee published Management Guideline of Immune Checkpoint Inhibitor-Related Toxicity in April 2019. This is the first local guideline focusing on immunotherapy, and using it, the prescribing physicians’ safety knowledge concerning such drugs will be enhanced, and those prescribers that called for training regarding treatment of adverse effects may transition into bold but knowledgeable medical oncologists. In addition, although inland indications for PD-1/PD-L1 inhibitors were approved later than they were abroad and there are less approved indications, most clinicians (77.9%) have prescribed the drug according to clinical research data and foreign indications. Such practical experience has the potential to make the criteria for off-label use clearer and to shed light on future directions of theoretical and clinical study. All the above findings indicate that exploring the usage of PD-1/PD-L1 inhibitors in China will undoubtedly promote the evolution of cancer immunotherapy in the country.

On the other hand, the fact that over 70% of respondents have never engaged in any ICI-related research hints that some conflicts exist that are worth discussing. The first issue appears to be the lack of understanding of the mechanisms and efficacy of PD-1/PD-L1 inhibitors, which was the most significant factor preventing some respondents from prescribing the drug. Second, there is extensive use of PD-1/PD-L1 inhibitors in off-label situations (77.9%). For instance, pembrolizumab is approved for metastatic melanoma and advanced NSCLC in China, while it is approved for classical Hodgkin lymphoma, metastatic nonsquamous NSCLC, metastatic urothelial carcinoma and metastatic gastric or gastroesophageal junction adenocarcinoma in America [17]. However, our survey suggests that actual applications of ICIs in China exceed the indications approved by the China Food and Drug Administration. Third, prescribers who have not participated in any relevant research are not qualified to “identify pseudoprogression and hyperprogression” (another one of the top two concerns regarding drug utilization); therefore, they usually have trouble judging efficacy and deciding whether to discontinue the treatment or switch to another ICI. Finally, a few prescribers (4.9%) did not accurately recognize the safety of the drug owing to the occasional occurrence of serious adverse effects, and this lack of recognition partly resulted from incomplete theoretical and clinical knowledge and applicable guidelines. The above deficiencies may not only impact the treatment and prognosis of patients with tumors but also delay the progress of one’s personal career as a medical oncologist.

To overcome such shortcomings, the relevant state department should establish an efficient permission regulation system for conducting immunotherapy and implement specific training and re-education for doctors. Pharmaceutical companies should increase their investment in clinical trials of immunotherapy and in the promotion of this work to civilians. Additionally, clinicians should consciously take steps to study the progress of theoretical and clinical research to further standardize their clinical operation regarding immunotherapy. In addition, CSCO IO, the leader and pioneer of cancer immunotherapy in China, is bound to play a larger role in helping Chinese physicians stay up to date on the newest information about ICIs by organizing city tours, publishing guidelines, holding national conferences, and sustaining cooperation with international organizations such as the American Society of Clinical Oncology (ASCO) and the European Society for Medical Oncology (ESMO). With the measures above, the number of prescribers will hopefully be further increased, and both the safety index during treatment and the countermeasures to adverse effects should also increase.

## Conclusion

In conclusion, the present study generated a clearer understanding of the current status of PD-1/PD-L1 inhibitor use in China, which will help us carry out more purposeful doctor re-education. The prescription of PD-1/PD-L1 inhibitors is already very popular with Chinese oncologists, and this popularity is a little sequacious. Not understanding the mechanism and efficacy is the major barrier for prescription of PD-1/PD-L1 inhibitors in Chinese tumor treatment-related departments. In addition, this survey has further clarified the issues that affect doctors’ clinical decisions and laid a solid foundation for subsequent corresponding clinical research. The problems that need to be addressed urgently are “how to identify pseudoprogression and hyperprogression” and “immunity-related adverse effect management”. Improved measures to address these issues will likely guarantee the safety and efficacy of ICI therapy in China. The biggest limitation of this survey was that only doctor groups were surveyed, and as such, there was little information about immunotherapy from the patient point of view. The CSCO IO should put forward a plan to illustrate the status of cancer immunotherapy in patient groups.

## Data Availability

The datasets used and/or analyzed during the current study are available from the corresponding author on reasonable request. In particular, this questionnaire used in your study has not previously been published elsewhere.

## Abbreviations

CFDA: China food and drug administration
CSCO IO: Chinese society of clinical oncology immuno-oncology
CTLA-4: Cytotoxic T lymphocyte-associated protein 4
FDA: Food and drug administration
ICI: Immune checkpoint inhibitor
NSCLC: Non-small cell lung carcinoma
PD-1: Programmed cell death 1
PD-L1: Programmed cell death ligand 1
RCC: Renal cell carcinoma
SCCHN: Squamous cell carcinoma of the head and neck
SCLC: Small cell lung cancer
